# Impact of hip abductor and adductor strength on dynamic balance and ankle biomechanics in young elite female basketball players

**DOI:** 10.1038/s41598-022-07454-3

**Published:** 2022-03-03

**Authors:** Fernando Domínguez-Navarro, Josep Carles Benitez-Martínez, Borja Ricart-Luna, Pedro Cotolí-Suárez, Jose María Blasco-Igual, Jose Casaña-Granell

**Affiliations:** 1 Department of Physiotherapy, University of Valencia, Calle Gascó Oliag 5, 46010 Valencia, Spain; 2 Department of Physiotherapy, Faculty of Health Sciences, European University of Valencia, 46010 Valencia, Spain; 3 I+D+I Alqueria LAB Department, Valencia Basket Club, Valencia, Spain; 4 Group in Physiotherapy of the Ageing Processes, Departament de Fisioteràpia, Universitat de València, Valencia, Spain; 5 Exercise Intervention for Health Research Group (EXINH-RG), Department of Physiotherapy, University of Valencia, 46010 Valencia, Spain

**Keywords:** Rehabilitation, Orthopaedics

## Abstract

This study aimed to evaluate, in an isolated and relative manner, hip abductor (ABD) and adductor (AD) strength and to study the extent to which these factors are related to balance and ankle dorsiflexion mobility in young elite female basketball players. Sixty trainee-level elite female basketball players (13–18 years old), who voluntarily agreed to participate in the study, were divided into three subgroups based on competition age divisions (U14, U16, U18). Isometric hip ABD and AD strength in each leg was evaluated using the *ForceFrame Strength Testing System*, also calculating the strength ratio and imbalance between legs. Y Balance Test (YBT) and ankle dorsiflexion mobility were also assessed. ANOVA was used for between-group differences analysis. Likewise, the impact of hip strength on balance and ankle mobility was analyzed using Pearson's correlation coefficient. A linear regression model for dependent variables was created with all variables that exhibited significant correlations. A between-group comparison analysis for the three competition age subgroups (U14, U16, U18) revealed non-significant differences (*p* > 0.005) for the hip strength variables except for hip ABD strength. The correlation study showed low-moderate effect sizes for hip ABD (in both the contralateral and homolateral limb) and AD strength (only the homolateral limb) with YBT and ankle dorsiflexion. However, when performing a regression model, only right hip ABD significantly predicted right limb YBT scores (β = 0.592, *p* = 0.048). The present study indicated that, although both hip ABD and AD strength correlate with balance and ankle mobility with low-moderate effect sizes, only hip ABD strength was found to significantly predict YBT scores. Therefore, the potential role of hip ABD strength in particular, but also hip AD strength, for monitoring and enhancing balance and ankle mobility outcomes, should be taken into consideration when designing and implementing preventive strategies for lower-limb injuries.

## Introduction

Over the last decades the number of female basketball players has grown enormously, both in senior and trainee categories^1^, becoming one of the most popular sports in Europe and the United States^2^. The greater number of participants also implies an increased number of injuries associated with this sport, so there is an increasing scientific interest in reporting and analyzing the most frequent injuries among female basketball players, as well as developing prevention strategies.

In this regard, the most common injuries reported affect the lower limb (LL) due to loss of balance in jumping and landing^3^. Specifically, ankle sprains and tears of the anterior cruciate ligament (ACL) are the two most prevalent injuries in this sport^2^, while the risk of suffering an ACL injury has been reported to be between two and eight times higher in female versus male basketball players^4^. Both types of injuries have a negative impact on the physical function and performance of players^5,6^, as well as significant economic consequences^7^_._

The functional condition of the hip muscles is considered a relevant factor in the risk of injury, and therefore, an important aspect to assess in female basketball players^8,9^. In addition, the study of the hip abductor (ABD) and adductor (AD) muscles is especially interesting, due to the essential role of these muscle groups in typical basketball movements, such as lateral displacements, changes of direction or single-leg balance situations^10^.

With regard to hip ABD muscles, some studies have suggested the impact of muscle strength^11^ or fatigue^12^ on LL-injury-related risk factors^13^. Precarious balance^14^ and altered ankle mobility^15^ may impact on risk of injury too. On the other hand, the role of AD muscles has been, in comparison, less studied. This evidence is mainly based on studies evaluating the influence of these muscles in sports performance and physical function^16^, there only being one study related to basketball population suggesting an association between AD shortening and altered coordination^17^ and balance.

Moreover, recent scientific research proposes evaluating hip ABD and AD strength not only alone, but also relatively^18^. Relative evaluation measures the strength of a muscle group in relation to the strength of antagonist muscles, namely, the ratio of hip AD:ABD strength; as well as in relation to the homologous muscles of the contralateral limb to assess possible asymmetries between legs^19^.

Although there are a number of studies related to different sport populations on the influence of hip ABD and AD muscles on balance and lower limb biomechanics, there is still nothing in the current literature evaluating the strength of these two muscles groups, in isolation and relatively, in female basketball players. We consider that such a study is necessary to establish the extent to which the strength of these two muscle groups affects aspects related to the risk of LL injury. Our hypothesis is that hip ABD and perhaps also hip AD strength correlate with balance and ankle mobility. The purpose of the study was to investigate to what extent hip ABD and AD strength, measured alone and relatively, are related to balance and ankle dorsiflexion mobility in young elite female basketball players.

## Methods

### Participants

This study took place at the premises of the Alquería del Basket, (Valencia, Spain) belonging to the Valencia Basket Club, during an off-season period (June and July 2020), coinciding with the Valencia Basket Female Summer Camp. A total of 60 trainee-level elite female basketball players (13–18 years old) agreed to take part in the study.

Basketball competition age divisions were used as subgroup allocation criteria, and therefore, three subgroups were created: U14 (seventeen participants), U16 (thirty-one participants) and U18 (twelve participants). Each subgroup consisted of players who were under the age of 14, 16 and 18 years old respectively at December 31st 2020. All the players belonged to First Division Spanish teams of women's basketball (Dia league). Players were excluded from the study in the following cases: (I) acute injury/condition that limited physical function; (II) lower limb injury within the past 6 months.

Participants and parents/legal guardians gave their written informed consent to both participate in the study and for publication of the images, in accordance with the ethical guidelines of the Declaration of Helsinki and subsequent updates. This study obtained the ethical approval of the Ethics Committee of the Universitat de València (number XXX). The research was developed and reported according to the recommendations of STrengthening the Reporting of OBservational studies in Epidemiology (STROBE)^20^.

### Procedure

First, the demographic and anthropometric data of the participants were collected. A standard 8-min warm-up was then performed (running, joint mobility exercises and neuromuscular activation exercises) prior to the recording of results derived from the following assessments: isometric strength of hip ABD and AD, the Y Balance Test (YBT) and the dorsiflexion Lunge test.

All tests were verbally explained and a first attempt for familiarization with the test was allowed without the results being recorded. Two members of the research team collected the data and then uploaded such data to a digital platform.

#### Hip strength procedure

**Figure 1:**
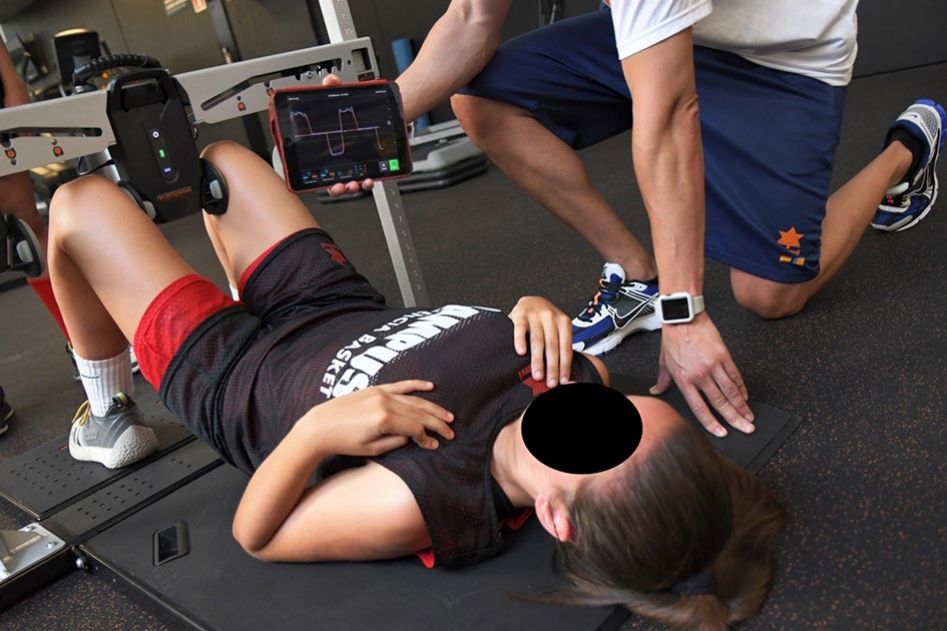
Measurement of hip adductor strength using the ForceFrame Strength Testing System.

**Figure 2:**
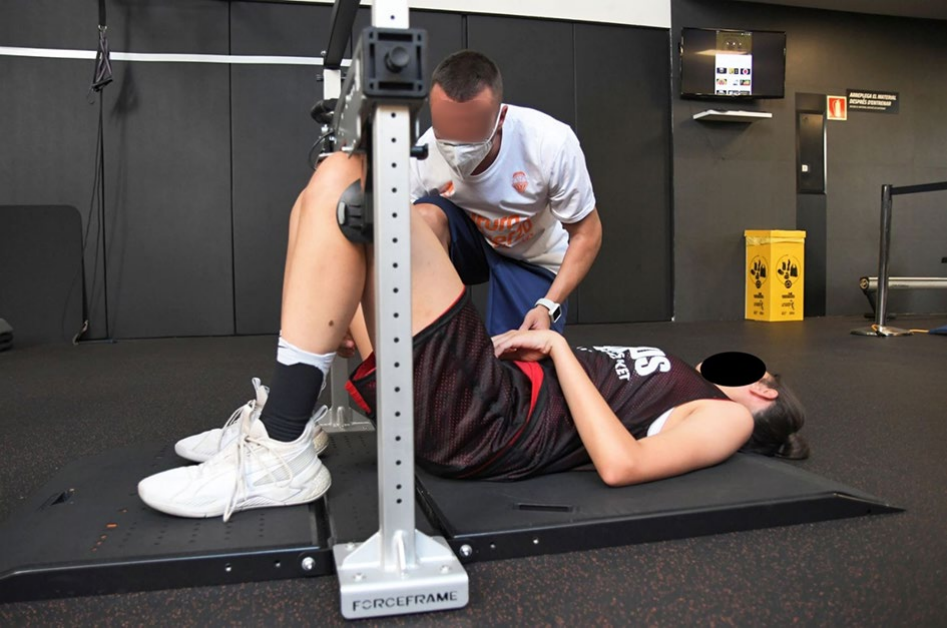
Measurement of hip abductor strength using the ForceFrame Strength Testing System.

The isometric strength of the hip ABD and AD muscles was collected using the ForceFrame Strength Testing System device (Vald Performance Albion, Australia), using the same protocol procedure as other previous studies^21^. For implementation, participants were asked to lie in a supine position with hips and knees bent 60º. The height of the bar was adjusted to each player to ensure that they maintained the angle. Participants were first asked to perform isometric contraction of the hip AD for 5 s and then, after a 5-s rest, a 5-s isometric contraction of the ABD (Figs. 1, 2). After a 45-s rest, the same procedure was repeated to be recorded or take the second attempt.

From the results obtained, two more parameters were calculated: the ratio of hip AD/ABD strength, calculated for each leg using the following formula: hip AD strength/hip ABD strength of the homolateral leg; and strength imbalance between limbs, both in the case of AD and ABD, the formula being: [(right leg muscle strength—left leg muscle strength)/right leg muscle strength] × 100.

#### Y balance test (YBT)

A functional test used to evaluate the dynamic balance, postural control and functional performance of the lower limbs from the sliding movement of one leg.

YBT is a simplified version of the Star Excursion Balance Test, whose modification involves analyzing the performance in only 3 of the 8 original directions: one, anterior; and the other two, aligned at 135º in the posterolateral and posteromedial directions respectively.

For the performance of the test, three lines of tape were placed on the floor. Participants were asked to move from an initial double-leg stance to single-leg stance while maximally reaching the multidirectional lines set with the opposite leg and lightly touching the tape with the distal end of the reaching foot, without compromising balance. The participants were barefoot to perform the test. The distance reached by the moving leg in the three dimensions was collected, and the balance score was calculated based on the sum of the three. The difference between the scores obtained for each leg was also calculated and expressed as a percentage.

The adapted YBT based on the original Star Excursion Balance Test appears as a simpler tool for evaluating functional performance and balance, while replicating the validity and reliability of the test^22^. In addition, the YBT has been used previously in other studies on basketball players^23^.

#### Weight-bearing ankle dorsiflexion test

The Lunge test was used to evaluate the dorsiflexion of the ankle joint in a weight-bearing position, according to Hoch and McKeon^24^, collecting the distance covered in centimeters.

A line of tape with metric marks (from 0 to 30 cm) was placed on the floor, perpendicular to the wall. Participants were asked to remain in a stride position in front of the wall, with both hands resting on the wall. The evaluated foot was placed forward, parallel to the tape and with the toe and heel touching the floor. The non-evaluated foot was placed comfortably behind the other foot with the knee extended. The participant took a stride with the forward knee flexed as much as possible anteriorly, with the heel of the evaluated foot touching the floor. A metal rod was used to determine the perpendicular projection of the knee to the floor and the distance reached was recorded. This method has reported excellent inter-rater (ICC = 0.82) and intra-rater (ICC = 0.88) reliability^25^.

Two members of the research team collected the data and then uploaded such data to a digital platform.

### Data management

Descriptive data included the mean and standard error of mean of the variables used. The normality of the data was verified by the Kolmogorov–Smirnov test. A comparison of means between the three competition age subgroups was preformed using ANOVA, and a Bonferroni test for post-hoc analysis The difference in means between the three groups for all variables evaluated using ANOVA was compared.

For the correlation analysis, the variables derived from the hip strength assessment, as well as anthropometric variables, were considered independent parameters, while YBT and ankle dorsiflexion mobility were chosen as dependent parameters. Pearson's R was used to explore the correlation of each of the dependent and independent parameters. Confidence intervals were set at 95%. The correlation effect size was interpreted as follows: < 0.1, trivial; 0.11–0.3, low; 0.31–0.5, moderate; 0.51–0.7, large; 071, − 0.9, very large; > 0.9, almost perfect^26^. A linear regression model for dependent variables was created with all variables that exhibited significant correlations. SPSS 24.0 software was used to perform statistical analysis^27^.

## Results

Table 1 shows the scores of all variables (classified as anthropometric, hip strength, balance, and ankle mobility variables) for the three competition age subgroups. Between-group comparison analysis revealed that height and weight significantly differ between groups (*p* < 0.001); whereas for the hip strength variables, there were no significant differences (*p* > 0.05) except for right and left hip ABD (*p* = 0.048, *p* = 0.030).

**Table 1:** Descriptive values and comparison study among age competition subgroups.

	U14 (n = 17)	U16 (n = 31)	U18 (n = 12)	Dif. intergroup
	Mean (SEM)	Mean (SEM)	Mean (SEM)	*p values*
**Anthropometric variables**				
Age (years)	13.4 (0.9)	15.2 (0.6)	17.3 (0.7)	
Height (m)	1.62 (0.07)	1.70. (0.07)	178.1 (0.06)	< 0.001*
Weight (kg)	53.7 (8.7)	59.9 (7.7)	71.3 (6.2)	< 0.001*
Dominant leg (n (percentage))				
Right	15 (88.2%)	27 (87.1%)	10 (83.3%)	0.243
Left	2 (11.8%)	4 (12.9%)	2 (16.7%)	0.581
**Hip strength variables**				
AD hip strength left leg (w)	226.0 (54.3)	233.2 (47.7)	236.8 (45.1)	0.881
AD hip strength right leg (w)	241.0 (59.3)	242.8 (46.4)	253.4 (45.2)	0.677
ABD hip strength left leg (w)	218.8 (55.7)	242.6 (36.0)	261.6 (40.1)	0.048*
ABD hip strength right leg (w)	208.1 (58.8)	234.6 (42.2)	246.3 (43.8)	0.030*
Inter-limb AD hip strength difference (%)	6.5 (5.2)	5.8 (4.4)	4.7 (2.6)	0.537
Inter-limb ABD hip strength difference (%)	7.3 (5.0)	6.1 (5.5)	6.2 (4.2)	0.734
AD/ABD ratio activation left leg (0 to 2)	1.0 (0.2)	1.0 (0.1)	1.0 (0.5)	0.488
AD/ABD ratio activation right leg (0 to 2)	1.2 (0.2)	1.1 (0.5)	0.9 (0.2)	0.137
**Balance and ankle mobility variables**				
Ankle DF test right leg	12.1 (2.3)	12.2 (2.9)	12.4 (3.3)	0.983
Ankle DF test left leg	12.6 (2.2)	12.4 (2.9)	12.5 (3.2)	0.774
Compound Y Balance Test left leg (cm)	79.2 (5.1)	82.9 (6.8)	91.7 (4.6)	0.369
Compound Y balance test right leg (cm)	76.6 (4.4)	81.7 (7.0)	89.6 (2.7)	0.647
Inter-limb difference in compound Y balance test (%)	4.6 3.9)	4.0 (2.4)	3.5 (2.2)	0.416

SEM: standard error of mean; w: watts DF: Dorsiflexion; AD: adductor; ABD: abdcutor.

As noted in Table 2, the correlation study revealed that both hip ABD and AD strength significantly correlated with YBT scores and ankle mobility. While hip ABD showed a trend to correlate with variables both in the homolateral and contralateral limb, hip AD exhibited significant correlations only with homolateral variables.

**Table 2:** Correlation study for the total sample of the study (n = 60).

	Ankle DF test left	Ankle DF test right	Compound Y balance test left	Compound Y balance test left right	Inter-limb difference in compound Y balance test
**Hip strength variables**					
Hip AD left	0.356 (0.005)*	0.237 (0.068)	0.275 (0.049)*	0.099 (0.451)	0.181 (190)
Hip AD right	0.221 (0.090)	0.267 (0.039)*	0.174 (0.184)	0.145 (0.631)	0.183 (0.185)
Hip ABD left	0.334 (0.007)*	0.331 (0.010)*	0.343 (0.007)*	0.295 (0.022)*	0.078 (0.577)
Hip ABD right	0.184 (0.159)	0.198 (0.129)	0.401 (0.001)*	0.348 (0.006)*	0.029 (0.835)
Inter-limb hip AD difference	− 0.170 (0.194)	− 0.088 (0.502)	− 0.109 (0.408)	0.044 (0.736)	− 0.165 (0.233)
Inter-limb Hip ABD difference	0.062 (0.639)	0.051 (0.697)	− 0.158 (0.227)	− 0.189 (0.148)	0.212 (0.123)
Ratio AD/ABD left	0.141 (0.281)	0.098 (0.456)	− 0.21 (0.872)	− 0.183 (0.162)	0.237 (0.084)
Ratio AD/ABD right	0.018 (0.889)	− 0.100 (0.446)	− 0.185 (0.156)	− 0.209 (0.119)	0.183 (0.185)
**Anthropometric variables**					
Age	− 0.006 (0.964)	0.020 (0.883)	0.046 (0.726)	0.018 (0.848)	0.074 (0.575)
Height	0.129 (0.349)	0.123 (0.370)	− 0.004 (0.973)	− 0.087 (0.513)	0.196 (0.137)
Weight	− 0.010 (0.944)	0.012 (0.927)	0.122 (0.355)	0.037 (0.779)	0.217 (0.096)

Pearson correlation (p values).

DF: dorsiflexion; AD: adductor; ABD: abdcutor. *Indicates statistically significant correlation (*p* < 0.05).

Significant values are in [bold].

Hip ABD strength significantly correlated with YBT scores both in the homolateral (right limb: Pearson = 0.348, *p* = 0.006; left limb: Pearson = 0.295, *p* = 0.022) and contralateral limb (right hip ABD and left YBT: Pearson_0.401, *p* = 0.001; left hip ABD and right YBT: Pearson = 0.343, *p* = 0.007), as well as left hip ABD with homolateral (Pearson = 0.334, *p* = 0.007) and contralateral (Pearson = 0.331, *p* = 0.010) ankle mobility.

For hip AD strength values, a significant correlation was observed for the left limb with ankle mobility (Pearson = 0.356, *p* = 0.005) and YBT Pearson = 0.275, *p* = 0.049), while right hip AD significantly correlated homolaterally with ankle mobility only (Pearson = 0.267, *p* = 0.039).

Table 3 shows the results for the predicting model scores; importantly, right hip ABD exhibited a significant predictor value for right YBT (β = 0.592, *p* = 0.048), while the rest of the variables showed non-significant differences.

**Table 3:** Predicting model values for dependent variables.

Dependent variable	R^2^ adj	Predictor	β	*p* value
Y balance test right	0.475	Hip ABD right	0.592	0.048*
		Hip ABD left	0.043	0.803
Y balance test left	0.255	Hip ABD right	− 0.205	0.575
		Hip ABD left	0.283	0.460
		Hip AD left	0.175	0.304
Ankle dorsiflexion right	0.010	Hip ABD left	− 0.060	0.735
		Hip AD right	0.055	0.843
Ankle dorsiflexion left	0.177	Hip ABD left	0.050	0.105
		Hip AD left	0.079	0.442

Significant values are in [bold].

## Discussion

The purpose of this study was to evaluate, in an isolated and relative manner, hip ABD and AD strength, and to study the extent to which these factors are related to balance and dorsiflexion mobility in young elite female basketball players.

Strength assessment should be part of a routine screening process for athletes, which may help to detect subjects with functional deficits, serving as a first step in the design of injury prevention strategies. Likewise, based on the physical performance demands in basketball^28^, the measurement of hip ABD and AD muscles should ideally be included in the strength assessment procedure.

The present study provides a comprehensive database for hip ABD and AD strength measured with the ForceFrame Strength Testing System, and combining isolated (right and left limb strength) with relative variables (inter-limb difference and ABD/AD ratio), this being, to the authors’ knowledge, a novel approach to express hip ABD and AD strength. In addition, those findings constitute a basis to further design specific interventions aimed at improving hip ABD and AD strength in that population.

The between-group mean comparison analysis revealed no significant differences in most of those variables among the three competition age subgroups, except for right and left hip ABD. Therefore, it reflects that only hip ABD strength increases as the basketball player gets older. Age was expected to be a determinant factor for hip strength values, and therefore, it would justify further analysis for each of the competition age subgroups individually. Maturational timing and menarche was thought to play an important role in this regard^29^. However, since most of the hip strength outcomes did not appear to be influenced by age, this hypothesis was not fully supported, and therefore, a separate analysis for each competition age subgroup was not needed.

The correlation study revealed a positive correlation between hip ABD and AD strength and balance and ankle mobility. However, the interpretation of these findings should be based on the regression model, according to which only hip ABD strength significantly predicted balance and ankle mobility outcomes. In particular, that predictive value was found between right hip ABD and right YBT.

Precarious balance^30^ and reduced ankle dorsiflexion mobility^15^ has been described in basketball players as a risk factor for LL injuries, such as anterior cruciate ligament injury and ankle sprain, these having the highest incidence among female basketball players^2^. Furthermore, there is solid evidence from several studies performed in different sport populations^31^ and in non-athletes^32^ postulating the importance of achieving adequate hip strength levels (especially hip ABD) to improve those outcomes and to reduce the risk of LL injuries. The results of the present study extend the knowledge on hip strength as a factor positively affecting balance and ankle mobility in female basketball players also, as evidence in this population was still scarce despite the high incidence of lower limb injuries. Focusing on the relation between hip ABD and YBT scores, Wilson BR et al.^33^ also found hip ABD strength to be the only variable evaluated significantly predicting YBT scores in a study involving 73 subjects; Lee et al*.*^34^ found abductor strength to correlate with moderate-large effect size with YBT scores in an adult female population. Hip abduction strength is proposed to be an important factor for performance in YBT, as the test requires a series of single-leg squat-like maneuvers.

With respect to hip AD, the evidence in basketball population was limited to having established that adductor shortening is correlated with altered coordination^17^. Thus, this trial contributes novel knowledge suggesting that this muscle strength may impact on balance and ankle mobility.

Interestingly, different correlation trends were found between hip ABD and AD. Hip ABD strength showed a significant correlation with parameters in both the homolateral and contralateral limb, while hip AD muscles only correlated with the homolateral limb.

We believe that this is due to the specific action of hip ABDs, especially the gluteus medius and the gluteus minimums when stabilizing the hip and pelvis, which allows the moving leg to optimize its movements while the pelvis remains stable^35^. However, an optimal functional status of both muscles, and not only the abductors, enables more efficient lower-limb patterns, which may have a positive influence on preventing lower-limb injuries.

Measuring muscle ratios is a relatively novel approach to express muscle strength levels, and, as proposed by some authors, a more predictive outcome for lower-limb injury incidence^18^. The quadriceps-hamstring ratio has been used as a measure to determine the risk of knee and ankle injuries^36^, but the strength ratio between adductor and abductor muscles has not been widely used for this purpose. Intriguingly, the current study found that this measure did not correlate with balance or ankle mobility. Studies evaluating AD/ABD ratio have only been carried out in ice hockey and football players^37,38^, so further investigation with female basketball players is needed to establish any potential impact of this outcome on functional and biomechanical outcomes, and thus, may be a relevant and valid tool to be included in a routine functional evaluation.

## Limitations

This study was carried out on a sample of 60 subjects, which may be considered sufficient, although it is true that a greater number of participants would make it more representative. On the other hand, only healthy subjects with no recent history of injury were included in this study, so data cannot be applied to those recovering from an injury. Another important consideration is that only one post-season assessment was made, so it is unknown if values might fluctuate throughout the season and how relevant this may be. Also, the impact of basketball practice on players' skill levels was not examined directly, and it may lead to individual differences in the organization and coordination of movements. The injury records of the participants have not been studied directly, so it could be convenient to take this into account for future studies in adult players. Lastly, these findings may only be applied to young trainee basketball players, and therefore, they cannot be extrapolated to senior players, which would require future studies.

## Conclusions

The present study, evaluating female basketball players aged 13–18 years old, reports that, although hip ABD and AD strength both correlate with balance and ankle mobility with low-moderate effect sizes, only hip ABD strength was found to significantly predict YBT scores. Accordingly, the potential importance of primary hip ABD strength, and also hip AD strength, for monitoring and enhancing balance and ankle mobility outcomes should be accounted for when designing and implementing lower-limb preventive strategies.

## Data Availability

Data will be fully available under request to the corresponding author.
